# Interleukin-33 regulates metabolic reprogramming of the retinal pigment epithelium in response to immune stressors

**DOI:** 10.1172/jci.insight.129429

**Published:** 2021-04-22

**Authors:** Louis M. Scott, Emma E. Vincent, Natalie Hudson, Chris Neal, Nicholas Jones, Ed C. Lavelle, Matthew Campbell, Andrew P. Halestrap, Andrew D. Dick, Sofia Theodoropoulou

**Affiliations:** 1 Academic Unit of Ophthalmology, Translational Health Sciences, Bristol Medical School,; 2 School of Cellular and Molecular Medicine, and; 3 Medical Research Council Integrative Epidemiology Unit, Population Health Sciences, Bristol Medical School, University of Bristol, Bristol, United Kingdom.; 4 Smurfit Institute of Genetics, School of Genetics and Microbiology, Trinity College Dublin, Dublin, Ireland.; 5 Wolfson Bioimaging Facility, University of Bristol, Bristol, United Kingdom.; 6 Institute of Life Science, Swansea University Medical School, Swansea, United Kingdom.; 7 Adjuvant Research Group, School of Biochemistry and Immunology, and Trinity Biomedical Sciences Institute, Trinity College Dublin, Dublin, Ireland.; 8 Department of Biochemistry, Bristol Medical School, University of Bristol, Bristol, United Kingdom.; 9 UCL Institute of Ophthalmology, University College London, London, United Kingdom.

**Keywords:** Metabolism, Ophthalmology, Glucose metabolism, Mitochondria, Retinopathy

## Abstract

It remains unresolved how retinal pigment epithelial cell metabolism is regulated following immune activation to maintain retinal homeostasis and retinal function. We exposed retinal pigment epithelium (RPE) to several stress signals, particularly Toll-like receptor stimulation, and uncovered an ability of RPE to adapt their metabolic preference on aerobic glycolysis or oxidative glucose metabolism in response to different immune stimuli. We have identified interleukin-33 (IL-33) as a key metabolic checkpoint that antagonizes the Warburg effect to ensure the functional stability of the RPE. The identification of IL-33 as a key regulator of mitochondrial metabolism suggests roles for the cytokine that go beyond its extracellular “alarmin” activities. IL-33 exerts control over mitochondrial respiration in RPE by facilitating oxidative pyruvate catabolism. We have also revealed that in the absence of IL-33, mitochondrial function declined and resultant bioenergetic switching was aligned with altered mitochondrial morphology. Our data not only shed new light on the molecular pathway of activation of mitochondrial respiration in RPE in response to immune stressors but also uncover a potentially novel role of nuclear intrinsic IL-33 as a metabolic checkpoint regulator.

## Introduction

The retina is referred to as an “immune-privileged” tissue, where immune homeostasis is maintained through hematopoietic immune cells, such as microglia, and immune-competent tissue-resident cells, such as the retinal pigment epithelium (RPE) (1). The RPE is highly differentiated and considered a postmitotic single-cell layer performing a host of functions critical to retinal homeostasis, including maintenance of the visual cycle and photoreceptor phagocytosis (2). RPE cells are highly metabolically active while providing critical metabolic support through, for example, directional transport of glucose and lactate to fuel the outer retina and particularly photoreceptors (3). Studies of cultured RPE cells show that they can derive energy from glucose by aerobic glycolysis or oxidative phosphorylation (OXPHOS), depending on culture conditions. Defects in RPE metabolic and mitochondrial function, associated with low-grade inflammation or age-related decline, are associated with retinal degenerative diseases, including age-related macular degeneration (AMD) (4). However, the causal link between RPE metabolic alterations and retinal function and disease remains unclear.

Immune cells with different functions use several metabolic pathways to generate adequate levels of energy stores to support survival and to produce numerous biosynthetic intermediates to enable cellular growth and proliferation (5). Understanding how immune-competent tissue-resident cells, like RPE, manage energy consumption to potentially support outer retinal function maintenance is crucial, yet unknown. RPE cells utilize reductive carboxylation (6) to support redox homeostasis. This in turn is reinforced by the disproportionate damage to mitochondrial DNA (mtDNA) in the RPE of individuals with AMD. Furthermore, the role of lipids, which accumulate in the macula, and their oxidation, has emerged as an important factor in AMD development (7).

IL-33, a member of the IL-1 family, is a type 2 cytokine that is constitutively expressed in the inner retina, RPE, and choroid (8), as well as in epithelial cells, endothelial cells, and fibroblasts (9). IL-33 functions as an “alarmin” molecule released from barrier cells following cellular damage (10). IL-33 is a pleiotropic cytokine with effects that are pro- or antiinflammatory depending on disease or cell context (11). This is reflected by major roles in infection, allergic responses, metabolic homeostasis, and cancer (12–14). Growing evidence suggests that IL-33 plays an important role in systemic metabolic diseases, like type 2 diabetes (15), and cardiac disease (16).

We have previously shown that TLR ligands (TLR-3, -4) induce upregulation of IL-33 in RPE cells without cell death. We also demonstrated a protective role for IL-33 in regulating ocular tissue responses to promote wound healing (8). Recent exploitation of the cytoplasmic hybrid technique for representative mtDNA haplotypes of AMD populations revealed alterations in IL-33 expression and altered bioenergetics (17). This, coupled with novel epigenetic roles for IL-33 as shown in the maintenance of mitochondrial function in adipose tissue (18), led us to investigate whether IL-33 acts as an intracellular checkpoint for RPE metabolism regulation. Here, we characterize RPE metabolic reprogramming in response to TLR ligation. We provide evidence that immune-mediated signals drive differential bioenergetic sourcing within the RPE, leading to a specific nuclear signature of IL-33 expression that maintains mitochondrial health and allows cells to respond and deliver energy intermediates required for RPE function.

## Results

### RPE has a differential metabolic response to varying TLR agonists.

The bioenergetic response of RPE cells to potentially harmful signals (through TLR stimulation) was investigated to ascertain the response and ability to maintain function under acute immune stress. We employed a Seahorse XF Analyzer (Agilent Technologies) to determine whether TLR activation altered RPE cellular metabolism.

**Figure 1:**
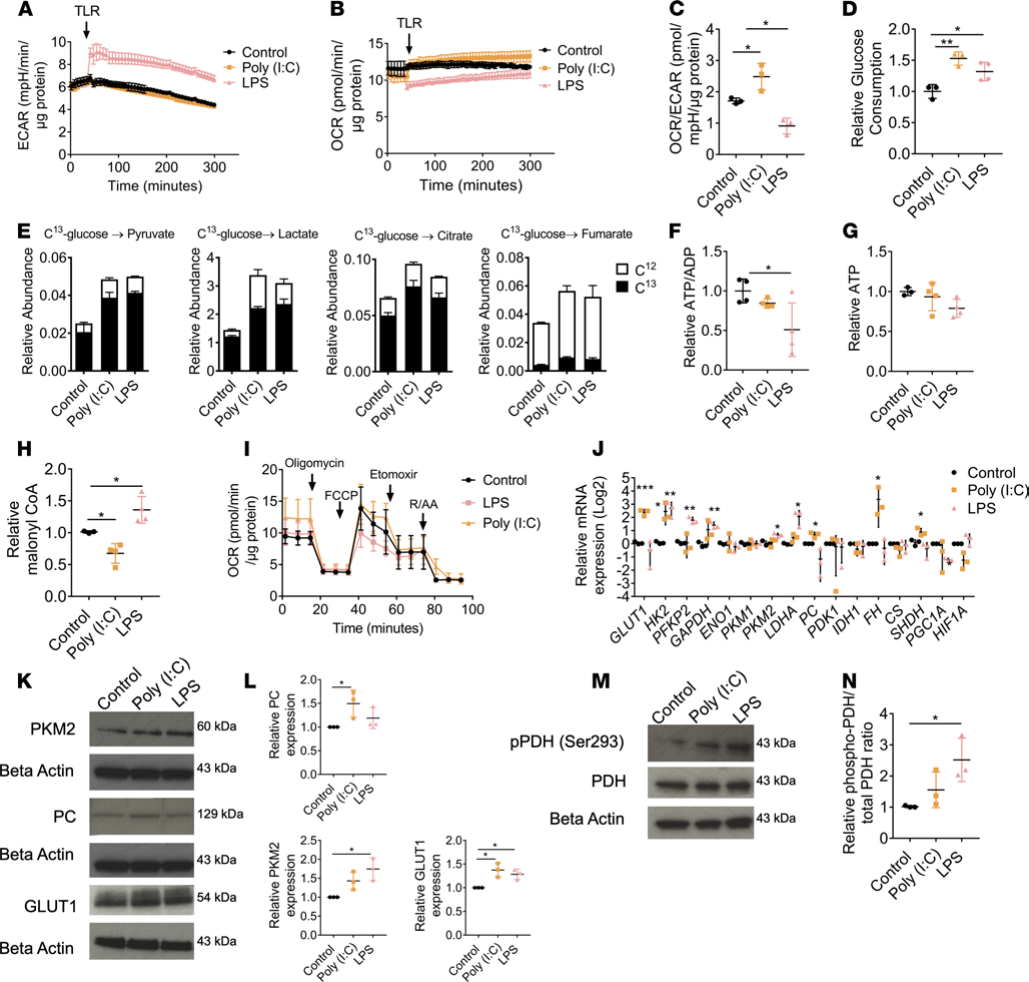
RPE has a differential metabolic response to varying TLR agonists. (**A**) ECAR changes following injection of LPS (1 μg/mL) or poly(I:C) (10 μg/mL) to ARPE-19 (*n* = 3). (**B**) OCR following injection of LPS or poly(I:C) to ARPE-19 (*n* = 3). (**C**) Relative OCR/ECAR ratio following treatment with LPS (30 minutes) or poly(I:C) (6 hours) to ARPE-19 (*n* = 3). (**D**) ARPE-19 treated for 24 hours with LPS or poly(I:C); glucose concentrations are expressed as relative consumption (*n* = 3). (**E**) Uniformly labeled C13-glucose incorporation into ARPE-19 metabolites treated for 24 hours with LPS or poly(I:C); relative abundance of C13 and C12 including pyruvate, lactate citrate, and fumarate (*n* = 3). ATP/ADP ratio (**F**) and relative ATP levels (**G**) of ARPE-19 stimulated for 24 hours with either LPS or poly(I:C) (*n* = 4). (**H**) Relative levels of malonyl CoA detected in whole cell lysates of ARPE-19 stimulated with either LPS or poly(I:C) for 24 hours (*n* = 3). (**I**) Modified mitochondrial stress test including a third injection of etomoxir (3 μg/mL) of ARPE-19 stimulated with either 30 minutes LPS or 6 hours poly(I:C) (*n* = 3). (**J**) ARPE-19 treated for 24 hours with LPS or poly(I:C); RNA was extracted and converted to cDNA; RT-PCR was used to determine the relative gene expression of targets involved in glycolysis or the TCA cycle (*n* = 3). GLUT1, glucose transporter 1; PC, pyruvate carboxylase; PKM2, pyruvate kinase M2. (**K**) ARPE-19 treated with LPS or poly(I:C) for 24 hours; protein was extracted and immunoblot analysis was used to determine the expression of PKM2, GLUT1, and PC. (**L**) Quantification of immunoblots presented in **K** (*n* = 3). (**M**) ARPE-19 treated with LPS (1 μg/mL) for 30 minutes or poly(I:C) (10 μg/mL) for 24 hours; protein was extracted and immunoblot analysis was used to determine the phosphorylation of PDH. (**N**) Quantification of immunoblots presented in **M** (*n* = 3). Data are expressed as means ± SD from at least 3 independent experiments. **A**–**C** and **I** represent the biological repeats from 3 independent experiments (*n* = 3); each biological repeat is the mean of 2 technical repeats (2 seahorse wells per experiment). One-way ANOVA with Dunnett’s multiple comparisons test; *P < 0.05, **P < 0.01, ***P < 0.001.

Stimulation of RPE by LPS, a potent ligand of TLR4, promoted a substantial increase (1.5-fold) in the extracellular acidification rate (ECAR) relative to resting controls (Figure 1A) accompanied by a decrease in oxygen consumption rate (OCR) in human RPE cells (ARPE-19) (Figure 1B). These data imply increased aerobic glycolysis in LPS-stimulated ARPE-19 cells (Figure 1C), and this was supported by an increase in glucose consumption (Figure 1D). In contrast to LPS, stimulation of RPE by poly(I:C), a ligand for TLR3, for 6 hours led to increased aerobic metabolism in TLR3-activated RPE (Figure 1C) accompanied by increased glycolytic reserve after oligomycin (Supplemental Figure 1, A and B; supplemental material available online with this article; https://doi.org/10.1172/jci.insight.129429DS1).

To confirm the differential bioenergetic adaptations of the RPE to TLR stimulation, abundance of intracellular metabolites stable isotope tracer analysis (SITA) was assessed with [U-13C]-glucose (schematic in Supplemental Figure 2A). SITA indicated that treatment with both LPS and poly(I:C) increased the incorporation of C13 glucose into glyceraldehyde-3-phosphate (G3P) (Supplemental Figure 2B), pyruvate, and lactate (Figure 1E), indicating increased glycolytic flux. Both treatments led to an increased incorporation of C13 glucose into TCA metabolites citrate and fumarate (Figure 1E) and succinate (Supplemental Figure 2C), aspartate (Supplemental Figure 2E), and glutamate (Supplemental Figure 2G). The increased labeled succinate and aspartate observed were attributed to an increase in the M+2 mass isotopologs (Supplemental Figure 2, D and F). Poly(I:C) treatment increased M+4 glutamate abundance (Supplemental Figure 2H), indicative of TCA cycling. Incorporation into the TCA intermediates was more pronounced with poly(I:C) treatment than LPS, confirming increased mitochondrial activity. Furthermore, the relative abundance of unlabeled C12 intermediates of fumarate, succinate, aspartate, and glutamate substantially increased with poly(I:C) treatment, suggesting other fuels may contribute to these metabolite pools (Figure 1E, Supplemental Figure 2C, and Supplemental Figure 2G).

Our data indicated increased glycolysis in LPS-stimulated ARPE-19 cells (Figure 1C), and this was supported by an increase in glucose consumption (Figure 1D). We further demonstrated that LPS, but not poly(I:C), decreased the ATP/ADP ratio in ARPE-19 cells (Figure 1F), indicating a switch away from mitochondrial metabolism. We also found that there were no significant alterations to ATP turnover in these cells following LPS treatment for 24 hours (Figure 1G).

In addition to glucose-derived carbon entering the TCA cycle, we investigated how fatty acid metabolism was affected by TLR signaling. First, we measured malonyl CoA levels in treated cells to assess fatty acid synthesis. We found that poly(I:C) stimulation led to a reduction of malonyl CoA, whereas LPS stimulation increased malonyl CoA levels (Figure 1H). We explored alterations to fatty acid oxidation through a modified mitochondrial stress test, including the third injection of etomoxir. We found that in response to LPS, fatty acid oxidation was reduced in RPE cells, whereas with poly(I:C) treatment, fatty acid oxidation was increased (Figure 1I).

Consistent with increased rates of glycolysis, reverse transcription PCR (RT-PCR) analysis demonstrated greater expression of glycolytic enzymes following LPS treatment (Figure 1J). Poly(I:C) treatment also increased expression of glycolytic enzymes, GLUT1, and TCA enzymes (Figure 1J). To confirm that the changes of gene expression were consistent with protein expression, immunoblotting was performed on 3 targets with significant alterations following TLR stimulation, GLUT1, PKM2, and PC, yielding comparable results (Figure 1, K and L).

Pyruvate dehydrogenase (PDH) is inactivated by the phosphorylation on a highly conserved serine residue (Ser293) in the E1 subunit that inhibits activity (19), restricting carbon entry into the TCA cycle. Consistent with the increased abundance of pyruvate and lactate, we observed that LPS treatment increased the phosphorylation, and thus inactivation, of PDH (Figure 1, M and N). Furthermore, inhibition of upstream kinase 1 (PDK1) reversed the LPS-mediated effects on ECAR and OCR (Supplemental Figure 1, C–F). Collectively, these data show that LPS stimulation of RPE drives a rapid switch to aerobic glycolysis, whereas poly(I:C) stimulation increases oxidative glucose metabolism and TCA activity.

We next investigated the structural changes to mitochondria that occur under innate immune activation. Mitochondria are hubs of metabolic activity, antiviral responses, and cell death cascades that continually remodel their structure to facilitate cell processes (20). Dynamic changes in mitochondrial remodeling are acutely responsive to changes in cell metabolism (21). Treatment with poly(I:C), but not LPS, increased mitochondrial size relative to unstimulated controls (Supplemental Figure 3, A and B). Additionally, there was shift in mitochondrial morphologies, with decreased fragmented mitochondria, and an increase in long and short tubular mitochondria observed with TLR stimulation (Supplemental Figure 3F).

Finally, we investigated whether the metabolic changes we observed were applicable to other RPE cells and not unique to this immortalized RPE cell line. We performed these experiments on primary murine RPE. Similar results were observed (Supplemental Figure 1, G–I). To determine whether the metabolic changes observed in RPE were cell specific, other TLR-expressing cells within the retina (human Müller glia and murine bone marrow–derived mast cells, BMMCs) were used as comparators. We observed a comparable metabolic response in Müller glia, whereas BMMCs increased their aerobic glycolysis in response to both agonists (Supplemental Figure 1, J–M).

### Alternate bioenergetic profiles are regulated by AMPK.

**Figure 2:**
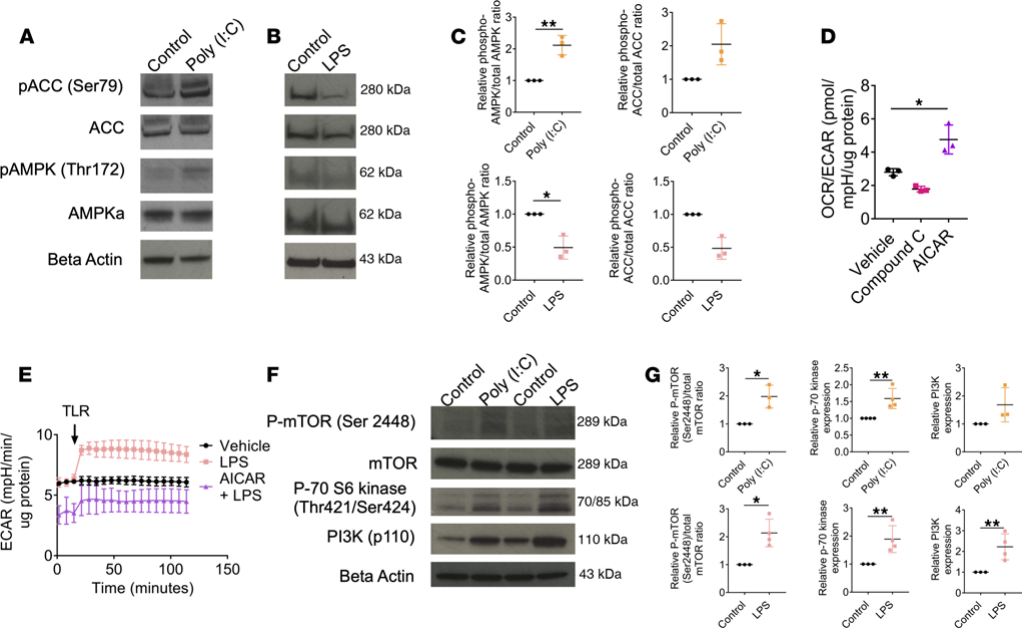
Alternate bioenergetic profiles are regulated by the activity of AMPK. (**A**) ARPE-19 were treated with poly(I:C) (10 μg/mL) for 6 hours; protein was extracted and immunoblot analysis was used to determine the phosphorylation of AMPK and ACC. (**B**) ARPE-19 were treated with LPS (1 μg/mL) for 30 minutes; protein was extracted and immunoblot analysis was used to determine the phosphorylation of AMPK and ACC. (**C**) Quantification of immunoblots presented in **A** and **B** (*n* = 3). (**D**) Relative basal OCR and ECAR of ARPE-19 treated for 30 minutes with either AMPK activator AICAR (1 mM) or inhibitor compound C (40 μM) (*n* = 3). (**E**) Real-time changes in relative ECAR following injection of LPS (1 μg/mL) to ARPE-19 cells ± AICAR (1 mM) pretreatment for 30 minutes (*n* = 3). (**F**) ARPE-19 were treated with poly(I:C) (10 μg/mL) for 6 hours or LPS (1 μg/mL) for 1 hour; protein was extracted and immunoblot analysis was used to determine the activation of mTOR, p-70 s6 kinase, and PI3K. (**G**) Quantification of immunoblots presented in **F** (*n* = 3). (**A**, **B**, and **F**) Data are expressed as means ± SD from 3 independent blots. (**D** and **E**) Represent the biological repeats from 3 independent experiments (*n* = 3); each biological repeat is the mean of 2 technical repeats (2 seahorse wells per experiment). (**D**) One-way ANOVA with Dunnett’s multiple comparisons test; *P < 0.05. (**E**) Unpaired Student’s *t* test; *P < 0.05, **P < 0.01.

Like immune cells (22), we hypothesized that in the RPE, TLR-induced metabolic responses are governed by AMPK activation status. ARPE-19 cells were cultured in the presence or absence of poly(I:C) or LPS at a range of time points, from 30 minutes to 24 hours. Treatment with poly(I:C) had a maximal effect at 24 hours, upregulating the activity of AMPK, demonstrated by measuring phosphorylation of its downstream target, acetyl-CoA carboxylase (ACC) (Figure 2, A and C). Stimulation with LPS reduced the phosphorylation of both AMPK and ACC observed at 30 minutes (Figure 2, B and C), supporting the metabolic changes shown in Figure 1, A and B.

Confirming its role as a key metabolic regulator, we found that pharmacological activation and inhibition of AMPK had opposing effects on RPE metabolism (Figure 2D and Supplemental Figure 4). The increase in OCR/ECAR with AICAR treatment supported the hypothesis that AMPK inhibits aerobic glycolysis (23). Further, we demonstrate that pharmacological activation of AMPK attenuated the switch to aerobic glycolysis in response to LPS (Figure 2E).

The downstream PI3K/mTOR signaling pathway was also examined following TLR activation in RPE cells. Poly(I:C) treatment increased mTOR phosphorylation on Ser2448 of the catalytic subunit and increased the expression of mTOR substrate/surrogate p-70 S6 kinase (Figure 2, F and G). Stimulation with LPS upregulated the expression of PI3K p110 (Figure 2, F and G). Downstream of PI3K, LPS treatment led to increased activity of mTOR and p-70 S6 kinase (Figure 2, F and G).

These data demonstrate that the differential metabolic responses to TLR stimulation in the RPE are regulated by AMPK activity. The activation of downstream signaling pathways supports the appropriate alterations of RPE metabolism that facilitate effector functions under stress.

### IL-33 increases mitochondrial metabolism in RPE.

We have previously shown that TLR agonists induce the expression of IL-33 in RPE cells, and this regulates cell responses and tissue homeostasis (8). By extrapolation from observations of the metabolic changes associated with cytokine signaling in other immune cells (24), we hypothesized that activation of RPE through the IL-33/ST2 axis also induces a metabolic change.

**Figure 3:**
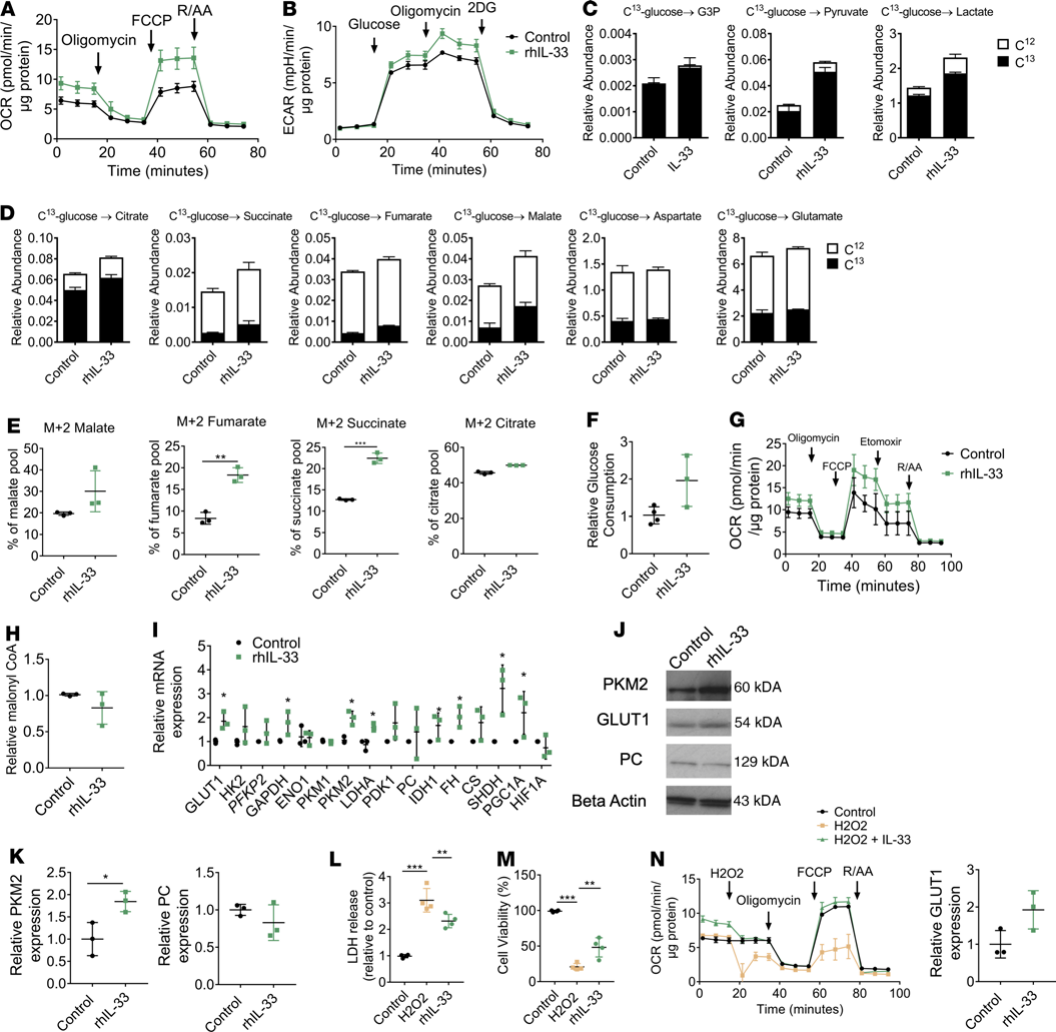
IL-33 increases bioenergetic demand. (**A**) Mitochondrial stress test from ARPE-19 treated with recombinant human IL-33 (rhIL-33) (100 ng/mL) 24 hours; XF injections were oligomycin (1 μM), carbonyl cyanide 4-(trifluoromethoxy)phenylhydrazone (FCCP) (0.5 μM), and rotenone/antimycin A (1 μM) (*n* = 3). (**B**) Glycolysis stress test from ARPE-19 treated with rhIL-33 (100 ng/mL) 24 hours; XF injections were glucose (10 mM), oligomycin (1 μM), and 2-deoxyglucose (100 mM) (*n* = 3). (**C**) Labeled C13-glucose incorporation into ARPE-19 glycolytic metabolites treated 24 hours with rhIL-33 (100 ng/mL) (*n* = 3). (**D**) Labeled C13-glucose incorporation into ARPE-19 TCA metabolites treated as in **C**. (**E**) Mass isotopomer distribution of C13-glucose–derived carbon into fumarate (M+2), succinate (M+2), and citrate (M+2) metabolite pools (*n* = 3). (**F**) ARPE-19 treated 24 hours with rhIL-33 (100 ng/mL); glucose concentrations were measured in the media prior/following treatment and expressed as relative consumption (*n* = 3). (**G**) Modified mitochondrial stress test including a third injection of etomoxir (3 μg/mL) of ARPE-19 cells treated 24 hours with rhIL-33 (100 ng/mL) (*n* = 3). (**H**) Relative levels of malonyl CoA detected in whole cell lysates of ARPE-19 (*n* = 3). ARPE-19 treated 24 hours with rhIL-33 (100ng/mL); (**I**) RT-PCR relative gene expression or (**J** and **K**) protein was extracted and immunoblot analysis was used to determine the expression of PKM2, GLUT1, and PC (*n* = 3). (**L**) ARPE-19 were treated with rhIL-33 (100 ng/mL) 12 hours before treatment with H_2_O_2_ (1 mM) 24 hours; LDH release was quantified in supernatants and expressed as relative to untreated control (*n* = 4). (**M**) ARPE-19 were treated with rhIL-33 (100 ng/mL) 12 hours before treatment with H2O2 (1 mM) 24 hours; an MTT assay was used to determine cell viability and expressed as a percentage of untreated control (*n* = 4). (**N**) Modified mitochondrial stress test of ARPE-19 treated 24 hours with rhIL-33 (100 ng/mL); XF injections were H_2_O_2_ (1 mM), oligomycin (1 μM), FCCP (0.5 μM), and rotenone/antimycin A (1 μM) (*n* = 3). Data are expressed as means ± SD from at least 3 independent experiments. (**A**, **B**, and **G**) Represent the biological repeats from 3 independent experiments (*n* = 3); each biological repeat is the mean of 2 technical repeats (2 seahorse wells/experiment). (**N**) Represents the biological repeats from 3 independent experiments (*n* = 3); each biological repeat is the mean of 2 technical repeats or single technical repeat (1 or 2 seahorse wells/experiment). One-way ANOVA, Dunnett’s multiple comparisons test; *P < 0.05,**P < 0.01, ***P < 0.005.

Alterations to the OCR/ECAR ratio were observed at 24 hours of treatment of ARPE-19 cells with IL-33 (Supplemental Figure 5, C and D), with no effect on RPE viability (Supplemental Figure 5, E and F). A mitochondrial stress test identified increases in oxidative metabolism following treatment, particularly increases in spare respiratory capacity (Figure 3A and Supplemental Figure 5G). Glycolytic metabolism was also increased (Figure 3B and Supplemental Figure 5H). Primary murine RPE also increased aerobic metabolism in response to recombinant IL-33 (rhIL-33) treatment in an IL-1-receptor like 1–dependent (IL-1RL1–dependent) manner (Supplemental Figure 5J).

SITA with [U-13C]-glucose indicated increased C13 incorporation into G3P, pyruvate, and lactate pools with IL-33 treatment (Figure 3C). We also observed increased C13 incorporation into TCA metabolites fumarate, malate, succinate, and citrate (Figure 3D). The distribution of the M+2 mass isotopolog was increased in the fumarate, malate, and succinate pools, following IL-33 treatment (Figure 3E). Glucose consumption was not significantly affected with IL-33 treatment (Figure 3F).

In addition to glucose-derived carbon entering the TCA cycle, we investigated how fatty acid metabolism was affected by IL-33 signaling. Consistent with the observed activation of AMPK/inactivation of ACC (Supplemental Figure 7, A–C), we observed an increase in fatty acid oxidation (Figure 3G). No changes were observed to the levels of malonyl CoA with IL-33 treatment (Figure 3H).

RT-PCR analysis further supported an increase in gene expression of glycolytic and TCA enzymes upon IL-33 stimulation (Figure 3I). Protein expression of PKM2, GLUT1, and PC correlated with gene expression analysis (Figure 3, J and K).

Given the functional significance of IL-33/ST2 signaling on mitochondrial capacity, we considered whether this pathway protects against oxidative damage to the RPE. To this end, H_2_O_2_ was used as an in vitro platform for inducing reactive oxygen species within the RPE (25). A dose-dependent relationship was observed between H_2_O_2_ concentration and cell viability (Supplemental Figure 6A). RPE cells treated with H_2_O_2_ for 24 hours exhibited reduced mitochondrial function and glycolytic capacity (Supplemental Figure 6, B and C). Pretreatment with IL-33 conferred some protection against H_2_O_2_-induced RPE dysfunction. This was indicated by reduced lactate dehydrogenase (LDH) release, an increase in cell viability to H_2_O_2_ alone (Figure 3, L and M), and maintenance of mitochondrial OXPHOS both at 12 and 24 hours (Figure 3N and Supplemental Figure 6D). IL-33 was unable to rescue the H_2_O_2_-mediated defects in glycolytic metabolism with 12 hours of treatment (Supplemental Figure 6E). Treatment with IL-33 for 24 hours had no significant initial “rescuing” effect to ECAR after H_2_O_2_, but at the greatest dose (100 ng/mL), ECAR levels were restored to controls by the end of the assay (Supplemental Figure 6F). When IL-33 was added at the same time as H_2_O_2_, there was no protective effect (Supplemental Figure 6G), likely due to oxidation and consequent inactivation of IL-33 (26). We could not attribute the effect of IL-33 treatment to expression changes in antioxidant enzymes or prosurvival proteins (Supplemental Figure 7, D–J). We observed that IL-33 induced a shift in mitochondrial morphology toward a more elongated phenotype and increased mitochondrial size (Supplemental Figure 8). Despite the observation that IL-33 increased PPARγ coactivator-1A (PGC1A), we found that IL-33 treatment had no significant effects on relative mtDNA expression levels (Supplemental Figure 8F), therefore suggesting that biogenesis may be unaffected (Supplemental Figure 8F).

### IL-33 is essential for the maintenance of mitochondrial respiration in RPE.

**Figure 4:**
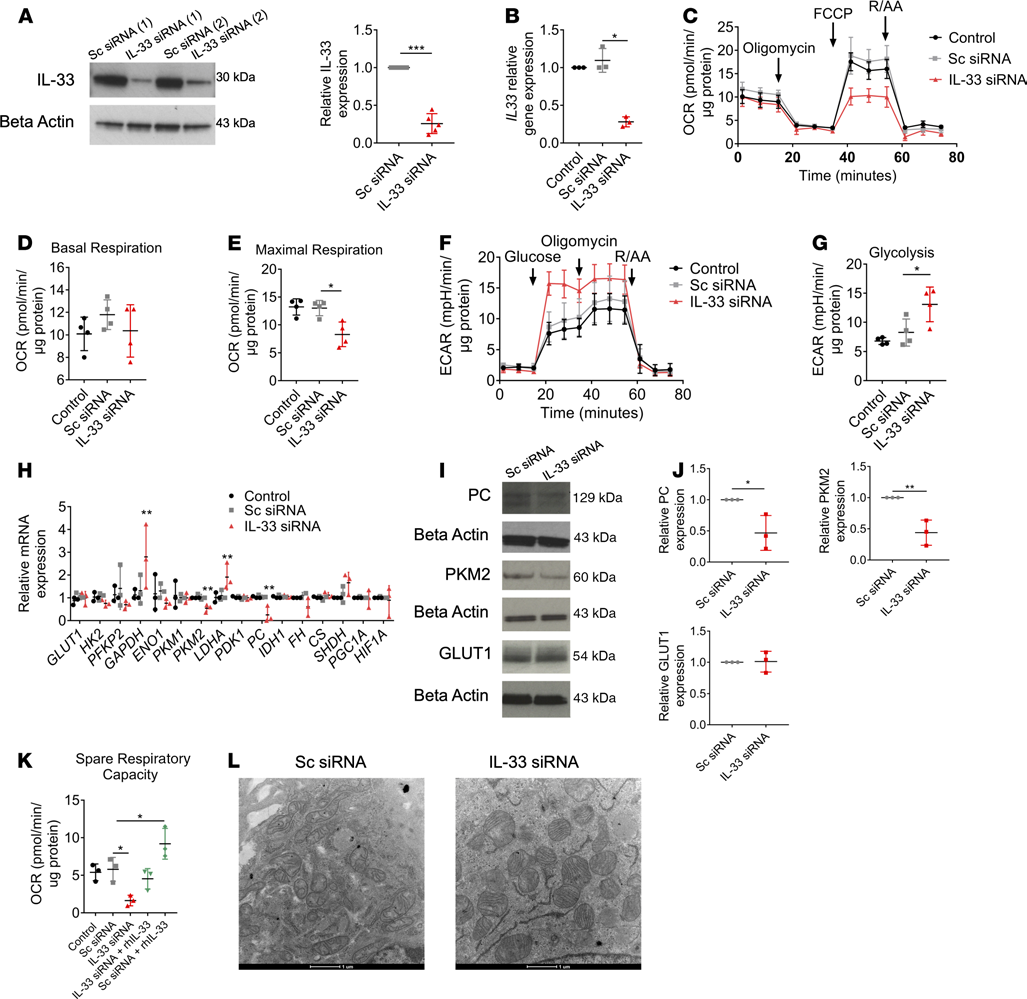
IL-33 is essential for the maintenance of mitochondrial respiration in RPE. (**A**) Immunoblot of IL-33 following transfection of ARPE-19 with IL-33 siRNA or scrambled siRNA (*n* = 5). (**B**) Gene expression of *IL33* following transfection of ARPE-19 with IL-33 siRNA or scrambled siRNA (*n* = 3). (**C**) Mitochondrial stress test following transfection of ARPE-19 with IL-33 siRNA or scrambled siRNA; XF injections were oligomycin (1 μM), FCCP (0.5 μM), and rotenone/antimycin A (1 μM) (*n* = 4). (**D** and **E**) Parameters calculated from **C** (*n* = 4). (**F**) Representative glycolysis stress test following transfection of ARPE-19 with IL-33 siRNA or scrambled siRNA; XF injections were glucose (10 mM), oligomycin (1 μM), and 2-deoxyglucose (100 mM) (*n* = 4). (**G**) Parameter calculated from **F** (*n* = 4). (**H**) ARPE-19 were transfected with IL-33 siRNA or scrambled siRNA; RT-PCR was used to determine the relative gene expression of targets involved in glycolysis or the TCA cycle (*n* = 3). (**I** and **J**) ARPE-19 cells were transfected with either IL-33 siRNA or scrambled siRNA; immunoblot analysis demonstrating expression of PKM2, GLUT1, and PC (*n* = 3). (**K**) Following transfection of ARPE-19 with IL-33 siRNA or scrambled siRNA, cells were treated with rhIL-33 for 24 hours; spare respiratory capacity was analyzed using a mitochondrial stress test; XF injections were oligomycin (1 μM), FCCP (0.5 μM) and rotenone/antimycin A (1 μM) (*n* = 3). (**L**) Representative transmission electron microscopy of ARPE-19 cells transfected with IL-33 siRNA or scrambled siRNA; original magnification, ×4500. Data are expressed as means ± SD from at least 3 independent experiments. (**C**–**G**) Represent the biological repeats from 3 independent experiments (*n* = 3); each biological repeat is the mean of 2 technical repeats (2 seahorse wells per experiment). (**K**) Represents the biological repeats from 3 independent experiments (*n* = 3); each biological repeat is either the mean of 2 technical repeats or a single technical repeat (1 or 2 seahorse wells per experiment). (**A**) Unpaired Student’s *t* test; *P < 0.05, ***P < 0.001. (**B**, **E**, and **G**–**K**) One-way ANOVA with Dunnett’s multiple comparisons test; *P < 0.05, **P < 0.01.

Recent studies have determined a novel role for IL-33 in the maintenance of mitochondrial respiration in beige and brown adipocytes (18). This coupled with the data we have shown thus far led us to further examine the effect of IL-33 loss on RPE bioenergetics. To knock down IL-33 in ARPE-19 cells, 4 preselected siRNA duplexes, each targeting different sequences of the human IL-33 gene, were utilized. The effectiveness of siRNA knockdown (KD) was confirmed at the protein and mRNA levels (Figure 4, A and B). Next, we examined the bioenergetic status using extracellular flux analysis. Mitochondrial stress analysis identified reduced maximal respiration compared with the scrambled siRNA, while no changes were observed in basal respiration (Figure 4, C–E). A glycolysis stress test identified increased glycolysis following IL-33 KD (Figure 4, F and G). The increased glycolysis observed with IL-33 depletion was confirmed by increased mRNA expression of key glycolytic enzymes lactate dehydrogenase A (LDHA) and GAPDH and a decrease in the key TCA enzyme PC (Figure 4H). Gene expression correlated to the protein expression of targets PKM2, GLUT1, and PC (Figure 4, I and J). The decreased OCR/ECAR ratio confirmed the increased aerobic glycolysis compared with mitochondrial activity (Supplemental Figure 9A). The addition of rhIL-33 to the IL-33KD cells was unable to recompense impaired mitochondrial function significantly (Figure 4K), indicating that it is mainly intrinsic IL-33 that regulates the metabolic responses we observed.

Given the marked reduction in mitochondrial metabolism in the absence of IL-33, we utilized transmission electron microscopy to identify any visible structural changes in IL-33KD RPE mitochondria. Compared with a scrambled siRNA, IL-33KD mitochondria appeared larger and more fragmented (Figure 4L). Image analysis of mitochondrial samples showed that mitochondrial diameter and area were increased with IL-33 siRNA and confirmed that a greater percentage of mitochondria were of a fragmented phenotype (Supplemental Figure 9B). The fragmented mitochondrial phenotype observed in the IL-33KD group was reminiscent of previously published data in IL-15 T memory cells, whereby mitochondrial fission promoted an increased glycolytic capacity (27).

### Bioenergetic analysis of IL-33^–/–^ primary RPE.

**Figure 5:**
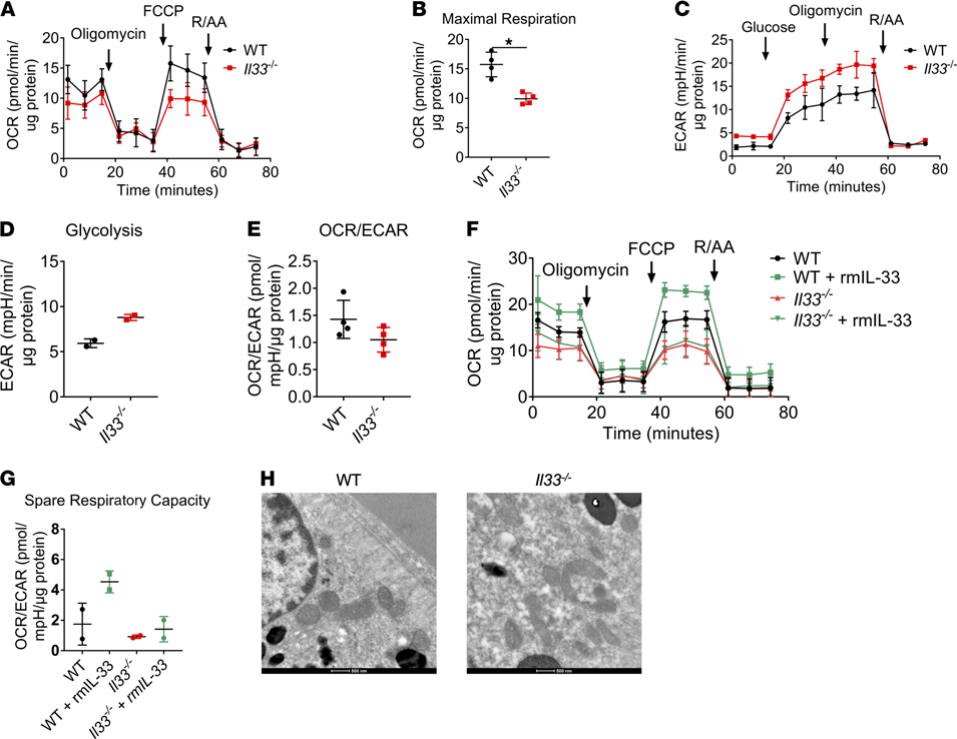
Bioenergetic analysis of Il-33^–/–^ primary RPE. (**A**) Mitochondrial stress test of WT and Il33^–/–^ primary murine RPE; XF injections were oligomycin (1 μM), FCCP (0.5 μM), and rotenone/antimycin A (1 μM) (*n* = 4). (**B**) Parameters calculated from **A** (as detailed in Results) (*n* = 4). (**C**) Glycolysis stress test of WT and Il33^–/–^ primary murine RPE; XF injections were glucose (10 mM), oligomycin (1 μM), and 2-deoxyglucose (100 mM) (*n* = 2). (**D**) Parameters calculated from **C** (as detailed in Methods) (*n* = 2). (**E**) OCR/ECAR ratio of WT and Il33^–/–^ primary murine RPE (*n* = 4). (**F**) Primary murine RPE cells were isolated from mice both WT and Il33^–/–^ mice and treated for 24 hours with rmIL-33; a mitochondrial stress test was used to assess mitochondrial function; XF injections were oligomycin (1 μM), FCCP (0.5 μM), and rotenone/antimycin A (1 μM) (*n* = 2). (**G**) Parameters calculated from **F** (as detailed in Methods) (*n* = 4). (**H**) Representative transmission electron microscopy of RPE from WT and Il33^–/–^ mice. Original magnification, ×9300. (**A**, **B**, and **E**) Represent the biological repeats from 4 independent experiments (*n* = 4); each biological repeat is either the mean of 2 technical repeats or a single technical repeat (1 or 2 seahorse wells per experiment). (**C**, **D**, **F**, and **G**) Represent the biological repeats from 2 independent experiments (*n* = 2); each biological repeat is either the mean of 2 technical repeats or a single technical repeat (1 or 2 seahorse wells per experiment). (**A**, **B**, and **E**) Represent data from 8 eyes (4 mice) per group. (**C**, **D**, **F**, and **G**) Represent data from 4 eyes (2 mice) per group. (**A** and **B**) Unpaired Student’s *t* test; *P < 0.05, **P < 0.01. (**F** and **G**) One-way ANOVA with Dunnett’s multiple comparisons test; *P < 0.05.

To investigate further the role of intrinsic IL-33, we performed a bioenergetic analysis of primary RPE derived from Il33^–/–^ mice. Using a mitochondrial stress test, we observed that in comparison with WT, Il33^–/–^ mice had reduced maximal respiration and spare respiratory capacity (Figure 5, A and B). No significant changes were observed in basal respiration or ATP production (Figure 5A). Like IL-33KD ARPE-19 cells, we observed that primary Il33^–/–^ RPE had increased glycolysis yet lacked glycolytic reserve measured after oligomycin treatment (Figure 5, C and D). There was no significant effect on the OCR/ECAR ratio (Figure 5E). Together, these results suggest that in the absence of IL-33 the relative contribution of glycolysis to ATP production compared with OXPHOS is greater. The addition of rmIL-33 to primary Il33^–/–^ RPE was unable to recompense impaired mitochondrial function (Figure 5, F and G).

Further supporting a nuclear role for IL-33, we observed that primary RPE derived from Il1rl1^–/–^ mice lacked any metabolic phenotype (Supplemental Figure 10). This excluded the possibility that metabolic alterations could have been attributed to lack of IL-33 release and autocrine/paracrine signaling through cognate receptor ST2.

We utilized transmission electron microscopy to identify any visible structural changes in Il33^–/–^ RPE mitochondria. Compared with WT, Il33^–/–^ mitochondria appeared smaller and more irregular in size (Figure 5H). Image analysis of mitochondrial samples showed that mitochondrial diameter was decreased in Il33^–/–^ (Supplemental Figure 11A). No significant changes were observed in mitochondrial area, but there was increased mitochondrial number/field in the Il33^–/–^ group (Supplemental Figure 11, B and C). In contrast to the data presented from IL-33 loss in human cells, image analysis identified an increase in “short tubular” mitochondria in the Il33^–/–^ group (Supplemental Figure 11D).

### Role of nuclear IL-33 in mitochondrial metabolism.

**Figure 6:**
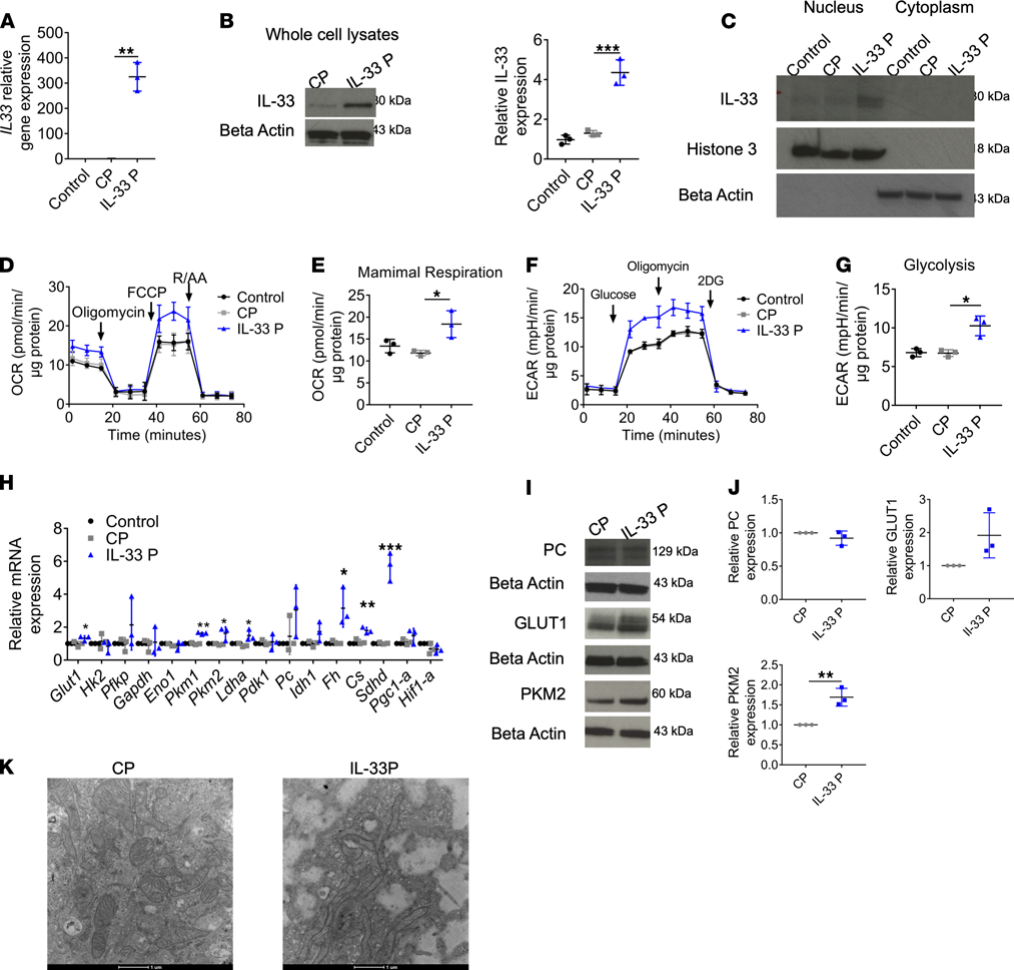
Role of nuclear IL-33 in mitochondrial metabolism. (**A**) Gene expression of IL33 following transfection of ARPE-19 with either an IL-33 activation plasmid or scrambled gRNA activation plasmid (*n* = 3). (**B**) Representative immunoblot of IL-33 expression in ARPE-19 transfected with either an IL-33 activation plasmid or scrambled gRNA activation plasmid (*n* = 3). (**C**) ARPE-19 were transfected with either an IL-33 activation plasmid or scrambled guide RNA (gRNA) activation plasmid; cell lysates were split into nuclear or cytoplasmic fractions, and Western blotting was used to determine the subcellular location of IL-33 (*n* = 2). (**D**) Mitochondrial stress test following transfection of ARPE-19 with either an IL-33 activation plasmid or scrambled gRNA activation plasmid; XF injections were oligomycin (1 μM), FCCP (0.5 μM), and rotenone/antimycin A (1 μM) (*n* = 3). (**E**) Parameters calculated from **D** (as detailed in Methods) (*n* = 3). (**F**) Glycolysis stress test following transfection of ARPE-19 with either an IL-33 activation plasmid or scrambled gRNA activation plasmid; XF injections were oligomycin (1 μM), FCCP (0.5 μM), and rotenone/antimycin A (1 μM) (*n* = 3). (**G**) Parameters calculated from **F** (as detailed in Methods) (*n* = 3). (**H**) ARPE-19 were transfected with either an IL-33 activation plasmid or scrambled gRNA activation plasmid; RT-PCR was used to determine the relative gene expression of targets involved in glycolysis or the TCA cycle (*n* = 3). (**I** and **J**) ARPE-19 were transfected with either an IL-33 activation plasmid or scrambled gRNA activation plasmid; expression of PKM2, GLUT1, and PC (*n* = 3) was determined by immunoblot. (**K**) Representative transmission electron microscopy of ARPE-19 cells transfected with an IL-33 activation plasmid or scrambled gRNA activation plasmid. Original magnification, ×4500. Data are expressed as means ± SD from at least 3 independent experiments. (**D**–**G**) Represent the biological repeats from 3 independent experiments (*n* = 3); each biological repeat is the mean of 2 technical repeats (2 seahorse wells per experiment). (**C**) Represents 2 independent immunoblots. One-way ANOVA with Dunnett’s multiple comparisons test; *P < 0.05, **P < 0.01, ***P < 0.001.

Following the identification of metabolic perturbations associated with IL-33 absence in vitro and ex vivo, we examined whether increased IL-33 expression in the RPE would have an adverse impact on cell metabolism. Using a CRISPR/Cas9 activation plasmid, the expression of the human IL-33 gene was observed to be upregulated in ARPE-19 cells. The effectiveness of CRISPR/Cas9-mediated overexpression of IL-33 was confirmed both at the RNA and protein level in whole cell lysates (Figure 6, A and B). Because IL-33 functions as a dual-function cytokine, residing within the nucleus and acting extracellularly (10), it was necessary to identify the subcellular location of IL-33 following overexpression. Increased IL-33 was observed in the nuclear subcellular fraction (Figure 6C). A mitochondrial stress test was used to identify changes in OXPHOS parameters associated with IL-33 overexpression, and this showed increased maximal (FCCP-induced) respiration rates (Figure 6, D and E). Glucose-starved cells were subjected to a glycolysis stress test to identify changes in glycolytic parameters, and IL-33 overexpression was found to significantly increase glycolytic metabolism (Figure 6, F and G). This was accompanied by increased gene expression of glycolytic and TCA cycle enzymes (Figure 6H). Protein expression of GLUT1, PC, and PKM2 was confirmed at the protein level (Figure 6, I and J). We finally confirmed alterations to mitochondrial function were accompanied by structural alterations. Image analysis indicated that mitochondria in the IL-33 overexpression group formed long elongated tubules (Figure 6K and Supplemental Figure 12), a structural phenotype associated with increased OXPHOS in T cells (27).

### Nuclear IL-33 promotes oxidative glucose metabolism.

**Figure 7:**
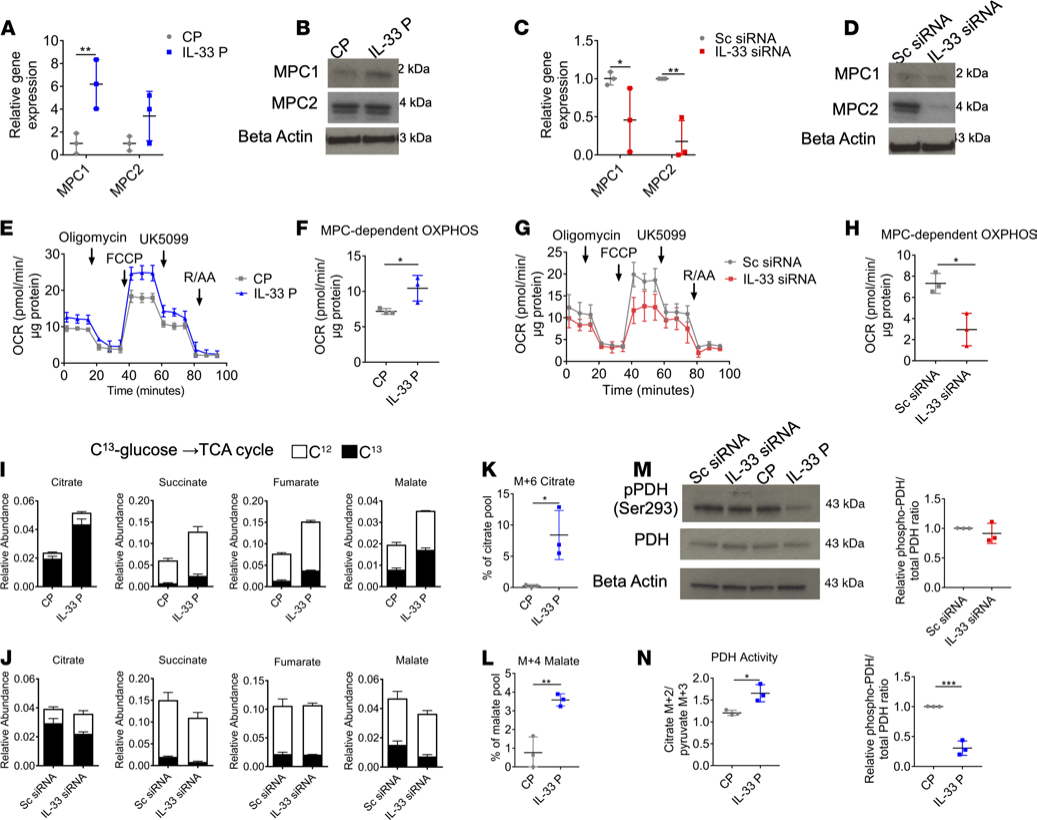
Nuclear IL-33 promotes oxidative glucose metabolism. (**A** and **B**) ARPE-19 were transfected with either an IL-33 activation plasmid or scrambled gRNA activation plasmid; (**A**) RNA was extracted, and RT-PCR was used to determine the expression of *MPC1* and *MPC2* (*n* = 3); (**B**) protein was extracted and Western blot analysis was used to determine the expression of MPC1 and MPC2 (*n* = 3). (**C** and **D**) ARPE-19 were transfected with either an IL-33 siRNA or scrambled siRNA; (**C**) RNA was extracted, and RT-PCR was used to determine the expression of *MPC1* and *MPC2* (*n* = 3); (**D**) protein was extracted and Western blot analysis was used to determine the expression of MPC1 and MPC2 (*n* = 3). (**E**) Modified mitochondrial stress test following transfection of ARPE-19 with either an IL-33 activation plasmid or scrambled gRNA activation plasmid; XF injections were oligomycin (1 μM), FCCP (0.5 μM), UK5099 (5 μM), and rotenone/antimycin A (1 μM) (*n* = 3). (**F**) Parameter calculated from **E** (as detailed in Methods) (*n* = 3). (**G**) Modified mitochondrial stress test following transfection of ARPE-19 with either an IL-33 siRNA or scrambled siRNA; XF injections were oligomycin (1 μM), FCCP (0.5 μM), UK5099 (5 μM), and rotenone/antimycin A (1 μM) (*n* = 3). (**H**) Parameter calculated from **G** (as detailed in Methods) (*n* = 3). (**I**) Uniformly labeled C13-glucose incorporation into ARPE-19 TCA cycle metabolites following transfection with either an IL-33 activation plasmid or a scrambled gRNA activation plasmid control; relative abundance of C13 and C12 including succinate, fumarate, malate, and citrate (*n* = 3). (**J**) Uniformly labeled C13-glucose incorporation into ARPE-19 TCA cycle metabolites following transfection with either an IL-33 siRNA or scrambled siRNA control (*n* = 3). Relative abundance of C13 and C12 including succinate, fumarate, malate, and citrate (*n* = 3). (**K** and **L**) Mass isotopolog distributions (MIDs) of ARPE-19 TCA cycle intermediates (**K**) M+6 citrate and (**L**) M+4 malate, following transfection with either an IL-33 activation plasmid or a scrambled gRNA activation plasmid (*n* = 3). (**M**) ARPE-19 were transfected with either an IL-33 activation plasmid/scrambled gRNA activation plasmid or an IL-33 siRNA/scrambled siRNA; Western blot analysis was used to determine the phosphorylation status of PDH (*n* = 3). (**N**) Citrate M+3/pyruvate M+3 ratio in ARPE-19 transfected with either an IL-33 activation plasmid or scrambled gRNA activation plasmid (*n* = 3). Data are expressed as means ± SD from at least 3 independent experiments. (**E**–**H**) Represent the biological repeats from 3 independent experiments (*n* = 3); each biological repeat is the mean of 3 technical repeats (3 seahorse wells per experiment). Unpaired Student’s *t* test; *P < 0.05, **P < 0.01, ***P < 0.001.

Because we observed an increase in ECAR (Figure 4, F and G) and extracellular lactate (Supplemental Figure 13A) in the absence of IL-33, we investigated whether IL-33 might regulate pyruvate import into the TCA cycle. Pyruvate enters the mitochondria through the mitochondrial pyruvate carrier complex (MPC) consisting of components MPC1/2 (28). Overexpression of IL-33 led to increased MPC1 expression, at mRNA and protein levels, but had no significant effect on MPC2 expression (Figure 7, A and B). IL-33 KD reduced the expression of both MPC components at the gene and protein levels (Figure 7, C and D). Hence, we performed a modified mitochondrial stress test with the additional injection of the MPC inhibitor UK5099 to assess the contribution of aerobic glucose metabolism to total OCR. In ARPE-19 cells with IL-33 overexpression, the increased maximal respiration was largely due to increased pyruvate-dependent respiration (Figure 7, E and F). When a similar experiment was performed on IL-33 siRNA cells, it was observed that pyruvate-dependent respiration had a significantly reduced contribution to maximal OCR (Figure 7, G and H).

To assess if pyruvate metabolism was the only component affected by the altered expression of IL-33, or if other metabolic pathways feeding into the TCA cycle were involved, a similar experiment was conducted to assess fatty acid oxidation (FAO). Etomoxir treatment after mitochondrial uncoupling significantly reduced the OCR; however, between control plasmid and IL-33 plasmid groups, this reduction was not significant (Supplemental Figure 13, D and E). With IL-33 loss, we observed that FAO was significantly increased (Supplemental Figure 13, F and G), suggesting that FAO is upregulated in the absence of IL-33 to compensate for defects in pyruvate metabolism.

SITA with [U-13C]-glucose was conducted to further assess how IL-33 altered glucose metabolism in RPE. In ARPE-19 cells with IL-33 overexpression, C13 labeling indicated increased glycolytic flux (Supplemental Figure 14A). No significant changes were observed in glycolysis “endpoint” metabolites pyruvate or lactate (Supplemental Figure 14A); however, substantial increases in C13 enrichment were observed in TCA metabolites (Figure 7I), indicating that glucose-derived TCA cycle activity was upregulated with IL-33 overexpression. In contrast, ARPE-19 cells with IL-33 KD had a significant increase in the abundance of C13 lactate (Supplemental Figure 14B) and decreased C13 enrichment in malate, citrate, and succinate (Figure 7J).

Overexpression of IL-33 led to an increase in the fully labeled citrate mass isotopolog (M+6) (Figure 7K). This pattern will occur in citrate when both oxaloacetate and malate are derived from glucose. Increased M+4 aspartate (Supplemental Figure 14C) and malate (Figure 7L) indicate the increased TCA cycling, which occurs with IL-33 overexpression. Labeling patterns using C13-labeled glucose then highlighted differential flux from glycolysis and pyruvate input into the TCA cycle. Glucose-derived pyruvate can enter the TCA cycle through PDH or PC (Supplemental Figure 14D). The citrate M+2/pyruvate M+3 ratio can serve as a surrogate for PDH activity, while the citrate M+3/pyruvate M+3 ratio is used as a surrogate of PC activity (29). IL-33 KD significantly reduced the citrate M+3/pyruvate M+3 (Supplemental Figure 14F) ratio, suggesting a decrease in the activity of the PC complex. Although no significant changes were observed in the citrate M+3/pyruvate M+3 ratio with IL-33 overexpression (Supplemental Figure 14G), there was a significant increase in the citrate M+2/pyruvate M+3 ratio (Figure 7N), suggesting that PDH activity was augmented with IL-33 plasmid treatment. Increased PDH activity was supported by reduced PDH E1 phosphorylation status (Figure 7M).

Glutamate labeling was unaffected by IL-33 overexpression (Supplemental Figure 15, A and B). However, we show that IL-33 knockdown reduced derived C13 labeling of the M+2 mass isotopolog in glutamate pools (Supplemental Figure 15, C and D). We found no observable C13 labeling detected in α-ketoglutarate pools (Supplemental Figure 15, E–H). The decrease in unlabeled α-ketoglutarate observed with IL-33 overexpression (Supplemental Figure 15E) suggests that the glutamine metabolism is likely reduced as increased glucose-derived carbon is used to support the TCA cycle. The increase in unlabeled glutamate and α-ketoglutarate (Supplemental Figure 15G) suggest that glutamine-derived carbon may support the TCA cycle when glucose metabolism is impaired.

Taken together these results indicate that nuclear IL-33 is a critical regulator of pyruvate oxidative metabolism in the RPE. When overexpressed, there is increased glycolytic flux into the TCA cycle most likely through increased MPC and PDH activity. The absence of IL-33 reduces the oxidative catabolism of glucose, and as pyruvate is “redirected” to lactate, FAO appears to support the TCA cycle.

## Discussion

All cells must be able to maintain their energetic resources to survive when quiescent and upon stress. The RPE provides an excellent model system to study basic cellular metabolism due to its active mitochondrial capacity, which maintains retinal homeostasis. RPE stress and metabolic alterations including mitochondrial dysfunction have been demonstrated to be implicated in retinal diseases (6, 30). However, the causal link between stress, metabolic alterations, and retinal degeneration remains unclear. In this study, we demonstrate that in response to stress, intrinsic cellular immune responses dependent on IL-33 act as a checkpoint for metabolic regulation. Control of this metabolic checkpoint means that the RPE can regulate metabolism for rapid energy production while ensuring the maintenance of mitochondrial health. We show here that the innate immune activation of the RPE through TLR signaling is supported by a distinct metabolic profile dependent on AMPK. We find that IL-33 signaling leads to broad transcriptional changes in metabolic genes, which leads to increased metabolic flux through glycolysis and the TCA cycle. Moreover, we provide evidence for a potentially novel role of intracellular IL-33 as a regulator of RPE metabolism. IL-33 loss increases aerobic glycolysis at the expense of oxidative glucose catabolism. Cells overexpressing IL-33 display increased expression of MPC1 while activating PDH (through dephosphorylation) to facilitate increased pyruvate flux into the TCA cycle.

The concept of a metabolic “retinal ecosystem” was recently introduced by Hurley et al. (3). They propose that energy homeostasis in the retina and RPE relies on a complex and specialized metabolic interplay between distinct cells, which has implications for retinal diseases (3). This study suggests that dysfunctional mitochondrial activity, acquired with age, restricts the concentration of glucose able to reach the photoreceptors and consequently reduces the lactate available to the RPE as a fuel source. This increased reliance on glycolysis may upset the metabolic ecosystem and lead to photoreceptor death. Our data support the proposal that AMPK, a central mediator of the bioenergetic response to innate immune stress, maintains energetic homeostasis in the RPE by increasing mitochondrial ATP production. We observed that a stressor, LPS stimulation of the RPE, inactivated AMPK. This elicited a metabolic shift toward aerobic glycolysis for ATP production, consistent with results observed in other immune cells, dendritic cells, and macrophages (22, 31). Interestingly, our data also provide evidence that TLR3 signaling in the RPE (a mechanism leading to retinal degeneration, ref. 32) is accompanied by the late activation of AMPK and increases in both glycolysis and OXPHOS. These data differ from results observed in dendritic cell populations whereby poly(I:C) stimulation drives increased aerobic glycolysis and a decrease in OXPHOS (33, 34), highlighting distinct properties of the RPE. Unlike AMPK, mTOR promotes catabolic and proliferative signaling and controls processes such as protein synthesis, metabolism, and cell growth that are essential for activated inflammatory cells (35). The activation of AMPK and mTOR alongside the increases in glycolysis and OXPHOS we observed following TLR stimulation in RPE supports the concept of a metabolic ecosystem essential for photoreceptor and neuronal function.

mtDNA can mediate expression levels of nuclear genes related to complement, inflammation, and apoptosis (17). Studies using cybrid RPE cells with representative mtDNA haplotypes demonstrated that different mtDNA variants exhibit a difference in ATP levels, lactate production, and metabolic (glycolytic) enzymes’ expression, which further dictates altered expression of nuclear encoded genes in complement, innate immunity, apoptosis, and proinflammatory signaling pathways (17). Our previous work demonstrated that the RPE responds to TLR agonists by increasing expression of IL-33. IL-33 is a nuclear cytokine whose expression correlates with mtDNA content in the RPE (8). Therefore, there are contemporaneous changes in RPE metabolism and expression of IL-33 when mtDNA content changes. We propose that IL-33 acts as a signal in the RPE to adapt or maintain mitochondrial metabolism. Consistent with prior reports on cancer cells (36) and fibroblasts (37), we have identified that IL-33/ST2 signaling is accompanied by increased metabolic (particularly glycolytic) activity. In addition, we found that exogenous IL-33 protects RPE cells against the noxious effects of oxidative stress. IL-33 release is regulated by oxidative stress and antioxidants within the airway epithelium (38). It is reported to reduce ROS production in fibroblasts (39) and directly enhance the activity of superoxide dismutase in cardiomyocytes (40). However, the data presented here demonstrate no change in antioxidant enzyme expression but rather an increase in metabolic flux and mitochondrial activity.

Our findings imply that IL-33 is essential for supporting mitochondrial respiration. We found that mice lacking IL-33 have abnormal mitochondrial morphologies. Upon subsequent in vitro investigation, we observed that endogenous IL-33 loss increases aerobic glycolysis as identified by increased lactate production from glucose. This increased reliance on glycolysis may therefore upset the metabolic ecosystem and lead to RPE dysfunction. This is consistent with observations in adipose tissue whereby isolated mitochondria from *Il33*^–/–^ mice exhibited profound respiratory defects, including reduced OXPHOS, and enzymatic activity of electron transport chain complexes II and IV (18). The same study identified an increased expression of genes involved in the catabolism of fatty acids, glucose, and amino acids in *Il33*^–/–^ adipose tissue, suggesting a similar compensatory mechanism in the face of reduced mitochondrial function (18). This highlights that IL-33 can have common roles across systems, and our data on its role in retinal metabolism can be extrapolated to other tissues and diseases.

The understanding of IL-33 has developed beyond the primary implications in the induction of Th2 immune responses to that of a cytokine with a broader activity in Th1 immunity and regulatory responses. In allergic and respiratory diseases, IL-33 activates type 2 innate lymphoid cells that elicit eosinophil recruitment and Th2 differentiation (41, 42). The proinflammatory role is supported by correlation of serum IL-33 with disease severity in atopic dermatitis (43) and through disease attenuation with IL-33 inhibition in murine allergic asthma (44). IL-33 exacerbates disease progression in rheumatoid arthritis models associated with proinflammatory cytokine production, mast cell degranulation, and neutrophil recruitment (45, 46). Contrarily, treatment with IL-33 reduces murine atherosclerosis development (16), and IL-33 polarizes macrophages (M2 phenotype), conferring protection against obesity and inflammation while improving glucose regulation.

Multiple lines of evidence suggest that increased aerobic glycolysis with IL-33 loss in the RPE occurs at the expense of oxidative glucose catabolism, as IL-33 impairs pyruvate import into the mitochondria through the MPC complex. Genetic support for this was obtained from IL-33KD RPE, where we found reduced expression of both MPC1 and MPC2 (Figure 7), which facilitate the transport of pyruvate into the mitochondria (47). Furthermore, cells overexpressing IL-33 displayed reduced aerobic glycolysis as mitochondrial activity was increased (Figure 6). The increased expression of MPC complex components and activity of PDH was accompanied by an increased consumption and oxidation of glucose within the TCA cycle. The increased spare respiratory capacity was found to be attributed to pyruvate import following the use of an MPC-specific inhibitor, UK5099 (48) (Figure 7E). We found that IL-33 is a key mediator of transcriptional changes to glycolytic and TCA cycle genes, which either drive the RPE toward aerobic glycolysis or mitochondrial catabolism of pyruvate in its absence or presence, respectively. Altered MPC1/2 expression results in significant metabolic disorders and has been previously shown to contribute to the Warburg effect in cancer cells (49).

While our data have uncovered a role for IL-33 in mitochondrial respiration in RPE, a key question that emerges from our work is how IL-33 regulates expression of MPC1/2 in RPE to facilitate the transport of pyruvate. The protective effect of exogenous rhIL-33 on RPE against oxidative stress was associated with increased metabolic flux and mitochondrial activity. In the future, generation of cell- and tissue-specific knockouts of IL-33 will be necessary to pinpoint the critical cell types that release IL-33 and the targets on which it acts to regulate the retinal metabolic ecosystem.

In conclusion, we have demonstrated that IL-33 constitutes a key metabolic checkpoint that antagonizes the Warburg effect to ensure the functional stability of the RPE. The identification of IL-33 as a key regulator of mitochondrial metabolism suggests roles for this cytokine that go beyond its extracellular “alarmin” activities. For example, when RPE is under stress, IL-33 contributes to minimize the effects of oxidative damage to the RPE and bolster mitochondrial metabolism. IL-33 exerts control over mitochondrial respiration in RPE by facilitating pyruvate import into mitochondria via upregulation of MPC expression and may be associated with the capacity of RPE to maintain homeostasis. Therefore, as well as identifying a molecular pathway for activation of mitochondrial respiration in RPE, our results demonstrate that intrinsic cellular IL-33 acts as a metabolic regulator exerting profound effects on retinal metabolism.

## Methods

### Cell lines

Immortalized human retinal pigment epithelium (ARPE-19) and human Müller glial Moorfields/Institute of Ophthalmology-Müller 1 (MIO-M1) cell lines were cultured in DMEM (4.5 mg/L) supplemented with 10% heat-inactivated FBS, 2 mM l-glutamine, 1 mM sodium-pyruvate, 0.5 μM 2-mercaptoethanol, 100 U/mL penicillin, and 100 μg/mL streptomycin. All cell lines were maintained at 37°C and were routinely screened for mycoplasma contamination. The human MIO-M1 was purchased from the UCL Business PLC. The ARPE-19 cell line is a spontaneously arising cell line derived from human RPE (ATCC number CRL-2302, ref. 50).

### Primary cells

C57BL/6J mice were purchased from Charles River Laboratories. All mice were maintained in the animal house facilities of the University of Bristol, according to Home Office Regulations. Animal husbandry and procedures complied with the Association for Research in Vision and Ophthalmology (ARVO) statement for the use of animals in ophthalmic and vision research. Il33^–/–^ mice were generated as described earlier (51) at the animal house facilities of Trinity College Dublin and sent to the animal house facilities of the University of Bristol when required. IL-1RL1 (ST2) knockout (Il1rl1^–/–^) C57BL/6 mice (backcrossed for 10 generations) were provided by C. Emanueli (School of Clinical Sciences, Bristol Heart Institute, University of Bristol, Bristol, United Kingdom) and were generated as described earlier (46).

Primary murine RPE cells were obtained as described earlier (52). Eyes were enucleated and incubated at 37°C in hyaluronidase for 45 minutes and in HBSS with calcium, magnesium, and 10 mM HEPES for a further 30 minutes. An incision was made beneath the ora serrata to remove the iris epithelium and cornea. The neural retina and the attachment to the optic nerve were removed. Eyecups were incubated at 37°C in trypsin EDTA for 45 minutes. Eyecups were then transferred into HBSS with 20% FBS and shaken gently to allow the RPE to detach. The RPE sheets were incubated at 37°C in 1 mL trypsin EDTA for 1 minute; 9 mL primary RPE media was then added, followed by centrifugation at 340*g* for 2 minutes at room temperature and supernatant removal. The resulting RPE cells were resuspended in 200 μL primary RPE media and homogenously mixed using a p200 pipette. Cells were used within 10 days of extraction. Cells were cultured in α-MEM, containing 1% N1 supplement, 1% glutamine-1% penicillin-streptomycin, 1% nonessential amino acid solution, 5% heat-inactivated FBS, 20 μg/L hydrocortisone, 250 mg/L taurine, and 13 ng/L triiodo-thyronin. Purity was assessed by immunoblotting for RPE-specific protein RPE65 and rhodopsin contamination from the neurosensory retina.

BMMCs were generated from C57BL/6 as previously described (53). Legs were taken from mice and the bones were cut at the end using angled scissors to expose the red marrow. Using a syringe and a 23-gauge needle, DMEM was flushed through the bone. DMEM containing the cell suspension was spun at 14,000*g* for 5 minutes in a centrifuge cooled to 4°C. The resulting cell pellet was resuspended in DMEM containing IL-3 (10 ng/mL) and dispensed into a cell culture flask. The cell suspension was cultured for 5 weeks to allow the generation of a mast cell population. Prior to functional assays, mast cells were sorted into a viable population using a MACS dead cell removal kit, and the purity was assessed by FACS based on CD117 and CD45 cell surface marker expression.

### Cell culture

Cells were seeded at a density of 100,000 per well of a 24-well plate and were exposed to different inflammatory stimuli: poly(I:C) (10 μg/mL), LPS (1 μg/mL), and IL-33 (1–100 ng/mL, as detailed in the results). Oxidative stress was induced by addition of hydrogen peroxide (1 mM). AMPK activity was modulated by activator AICAR (1 mM) and inhibitor compound C (40 μM) (Tocris Biosciences, Bio-Techne). Unstimulated controls and vehicle controls (DMSO) were always included.

### Western blot

Following treatment, protein extraction was performed using Cell-Lytic-M with the addition of Protease/Phosphatase Inhibitor Cocktail (MilliporeSigma). A commercial (Active Motif) nuclear extraction kit was used for the preparation of nuclear and cytoplasmic extracts as per the manufacturer’s instructions. Cells were washed twice with 1 mL of ice-cold PBS/phosphatase inhibitors, and 0.3 mL of ice-cold PBS/phosphatase inhibitors was added. Cells were removed from the cell culture plate by gentle scraping. Samples were pooled from 2 wells of a 24-well plate. The resulting cell suspension was centrifuged for 5 minutes at 200*g* in a centrifuge cooled to 4°C. The supernatant was removed and the pellet kept on ice prior to resuspension in 100 μL hypotonic buffer containing 5% (v/v) detergent. The resulting cell suspension was centrifuged for 30 seconds at 14,000*g* in a centrifuge cooled to 4°C. The supernatant was removed as the cytoplasmic fraction. The remaining pellet was resuspended in 25 μL of complete lysis buffer and incubated for 30 minutes on ice on a rocking platform. The resulting cell suspension was centrifuged for 10 minutes at 14,000*g* in a centrifuge cooled to 4°C. The supernatant was removed as the nuclear fraction.

Protein concentration was assessed using a BCA assay kit (Thermo Fisher Scientific). A total of 15 μg of protein was separated by SDS-PAGE, transferred to a PVDF membrane, and blocked in 5% milk/TBS/Tween-20. Immunoblotting was performed by the addition of a primary antibody against the protein of interest (antibody details provided in Supplemental Table 1). Proteins were detected with a polyclonal HRP-conjugated secondary antibody and visualized using chemiluminescent detection. Relative protein expression was calculated by normalization to β-actin, β-tubulin, or histone 3.

### RT-PCR

RNA was isolated from cells using the RNeasy extraction kit (QIAGEN). Purity and RNA concentration were assessed using a NanoDrop 3000 spectrophotometer (Thermo Fisher Scientific); 1 μg RNA was reverse transcribed using a SuperScript III First-Strand Synthesis system (Thermo Fisher Scientific). Resulting cDNA was then diluted 1:20 and amplified using SYBR Green reagents in a StepOne Plus detection system (Thermo Fisher Scientific). Cycling conditions: heat ramp 95°C for 10 minutes, extension (95°C for 15 seconds, 60°C for 1 minute) for 45 cycles, melt curve 95°C for 15 seconds, 60°C for 1 minute, 95°C for 15 seconds. Relative gene expression was calculated by normalization to β-actin. The primer sequences used are detailed in Supplemental Table 1.

### Genetic modulation of IL-33

Knockdown of IL-33 from ARPE-19 cells was achieved using the fast-forward transfection technique. Cells were seeded at a concentration of 55,000 per well of a 24-well plate in 0.5 mL of culture medium with 1% FCS and no antibiotics. Cells were incubated for 1 hour at 37°C prior to transfection. The FlexiTube GeneSolution (QIAGEN), as a specific mixture of 4 preselected siRNA duplexes, was used to target different sequences of the human IL-33 gene. Each siRNA was diluted in 100 μL of culture medium without serum and antibiotics (final concentration 20 nM each siRNA). HiPerfect transfection reagent (6 μL) was added to the siRNA, which was then vortexed and left for 5 minutes. Transfection complex (OriGene) (100 μL) was added to the cells and left for 48 hours at 37°C.

For IL-33 overexpression in ARPE-19 cells, a CRISPR/Cas9 activation plasmid was used to upregulate the expression of the human IL-33 gene. Cells were seeded at a concentration of 40,000 per well of a 24-well plate in 0.5 mL of culture medium with 10% FCS and no antibiotics. Cells were incubated overnight at 37°C prior to transfection. Medium was replaced just before transfection. For each transfection, 0.16 μg of plasmid DNA was diluted into 25 μL plasmid transfection medium. Separately, 0.833 μL of transfection reagent was diluted in 25 μL plasmid transfection medium. Both solutions were left for 5 minutes before being combined, mixed, and left for a further 30 minutes. Transfection complex (50 μL) was added to the cells and left for 48 hours at 37°C.

### Cell viability assays

Cell proliferation was assessed using the colorimetric MTT assay as per the manufacturer’s instructions (MilliporeSigma). A total of 10 μL of the 12 mM MTT stock solution in 100 μL of culture medium was added to cells and incubated at 37°C for 2 hours. All but 25 μL of medium was aspirated from the wells. DMSO (50 μL) was added and incubated 37°C for 30 minutes; the solution was transferred into a 96-well plate. Absorbances were read at 540 nm and expressed as a percentage of untreated control.

Cellular cytotoxicity was quantified using an LDH detection kit as per the manufacturer’s instructions. Briefly, 50 μL of cell culture supernatant was transferred to a 96-well plate with 50 μL of the reaction mixture and incubated 37°C for 30 minutes. Absorbances were read at 450 nm and expressed as relative to untreated control.

### Enzyme-based metabolic assays

Glucose consumption was assessed using a glucose coulometric assay kit as per the manufacturer’s instructions (Cayman Chemicals). A total of 15 μL of cell culture supernatant was transferred to a 96-well plate with 85 μL of diluted assay buffer. Then 100 μL of enzyme buffer was added and incubated at 37°C for 10 minutes. Absorbances were read at 520 nm. Glucose consumption was calculated by subtracting the glucose concentration of samples from the glucose concentration of unconditioned media and expressed as relative to an untreated control.

Extracellular lactate production was measured using an l-lactate fluorometric assay kit as per the manufacturer’s instructions (Cayman Chemicals). A total of 20 μL of cell culture supernatant was transferred to a 96-well plate with 100 μL diluted assay buffer, 20 μL of cofactor mixture, 40 μL of enzyme mixture, and 20 μL of fluorometric substrate. The plate was incubated for 20 minutes at room temperature and read using an excitation wavelength at 530 nm and an emission wavelength of 590 nm. Lactate production was calculated by subtracting the lactate concentration of samples from the lactate concentration of unconditioned media and expressed as relative to an untreated control.

ATP and ADP measurements were quantified using an ATP/ADP fluorometric assay kit (Cayman Chemicals). Cell supernatants were removed and 500 μL of nucleotide releasing buffer was added to cells. A total of 90 μL of this buffer was transferred to a 96-well plate and background ATP fluorescence was measured. Then 10 μL of ATP-monitoring enzyme was added and left for 2 minutes at room temperature. ATP fluorescence levels were then measured. The fluorescence levels were subsequently measured again to give background ADP fluorescence. Then 10 μL of ADP converting enzyme was added and left for 2 minutes at room temperature. ADP fluorescence levels were then measured. ATP and ADP levels were calculated and expressed as the ATP/ADP ratio. Data are expressed as relative to an untreated control.

### ELISA

Cell culture supernatants were analyzed for malonyl CoA using a commercially available ELISA kit (CUSABIO).

### Extracellular flux analysis

Cell metabolism was assessed using a Seahorse XFp Extracellular Flux Analyzer (Agilent Technologies). ARPE-19 and M1-MO were seeded at a density of 30,000 cells per well, 24 hours prior to analysis, with further treatments detailed in Results. Cell-Tak (Corning) was used to attach primary RPE and BMMCs at a density of 50,000 to the XFp. Real-time measurements of OCR and ECAR were normalized to total protein content using a BCA assay. Preoptimized injections specific for each assay were used. Cell Mito Stress kit (Agilent Technologies) injections: oligomycin (1 μM), FCCP (0.5 μM), and antimycin A/rotenone (1 μM). Glycolysis stress injections: glucose (10 mM), oligomycin (1 μM), and 2-deoxyglucose (2-DG) (1 μM). Glycolysis rate injections: antimycin A/rotenone (1 μM) and 2-DG (1 μM). UK5099 (5 μM) was used to inhibit the mitochondrial pyruvate transporter MPC1. Etomoxir (3 μM) was used to inhibit carnitine palmitoytransferase 1 (CPT1). GSK 2837808A (1 μM) was used to inhibit LDHA. Potassium dichloroacetate (25 mM) was used to inhibit PDK1.

XF medium included 25 mM glucose, 1 mM pyruvate, and 2 mM glutamine in minimal DMEM at pH 7.4. Metabolic parameters were calculated using the following formulae: OCR/ECAR (first OCR measurement)/(first ECAR measurement), proton leak (difference between OCR after oligomycin and nonmitochondrial respiration), nonmitochondrial respiration (OCR measurement after antimycin A/rotenone), basal respiration (difference between OCR before oligomycin and nonmitochondrial respiration), ATP production (difference between basal respiration and proton leak), maximal respiration (difference between maximum OCR after FCCP injection and nonmitochondrial respiration), spare respiratory capacity ([maximal respiration/basal respiration] × 100), glycolysis (difference between maximum ECAR before oligomycin and nonglycolytic acidification), glycolytic capacity (difference between maximum ECAR after oligomycin and nonglycolytic acidification), glycolytic reserve (difference between glycolytic capacity and glycolysis), nonglycolytic acidification (last ECAR measurement before glucose injection), basal glycolysis (last glycolytic proton efflux rate [glycoPER] measurement before rotenone/antimycin A), compensatory glycolysis (maximum glycoPER measurement after rotenone/antimycin A), mitoOCR/glycoPER ([(last OCR before rotenone/antimycin A) – (minimum OCR after rotenone/antmycin A)]/basal glycolysis), percentage PER from glycolysis ([basal glycolysis/basal PER] × 100), MPC-dependent respiration (difference between OCR before and after UK5099), and CPT1-dependent respiration (difference between OCR before and after etomoxir).

### Mass spectrometry

Gas chromatography coupled to mass spectrometry was performed using previously detailed methods (54). For SITA experiments, ARPE-19 cells were seeded at a concentration of 1,000,000 per well of a 6-well plate in 3 mL of normal culture medium with 10% FCS and antibiotics and left for 24 hours to reach subconfluence before treatment. Cells were washed twice with PBS before pulsing with [U-13C]-glucose medium for 2 hours. SITA media contained 10 mM [U-13C]-glucose, 2 mM glutamine, and 10% dialyzed FBS. Cells were washed twice with saline and lysed with 0.8 mL methanol, sonicated, and freeze dried using a speed vacuum. MIDs were derived using an algorithm developed at McGill University (55). Relative metabolite levels were normalized to protein content (μg) using a BCA assay.

### Electron microscopy

For ex vivo electron microscopy (EM), WT and Il-33^–/–^ mouse eyes were enucleated with the anterior chamber removed and fixed in 2.5% glutaraldehyde in 0.1 M phosphate buffer. Eyes were washed in buffer after fixation in osmium tetroxide for 1 hour and en bloc stained with uranyl acetate prior to being dehydrated with ethanol and embedded in Epon resin (TAAB labs). After polymerization the embedded eyes were trimmed to remove excess material, revealing the retina and choroid layers. These were sectioned transversely at 0.5 μm for light microscopy. Suitable areas were selected for transmission electron microscopy, and these were sectioned (80 nm thick) and stained with uranyl acetate and lead citrate prior to observation in a Tecnai 12 microscope. For in vitro EM, cells were grown to subconfluence in 24-well plates and treated as detailed in the results. Cells were washed in buffer and fixed in phosphate-buffered glutaraldehyde and postfixed using osmium tetroxide in the same buffer. Cells were stained with uranyl acetate and then after ethanol dehydration were infiltrated with Epon resin mix and polymerized at 60°C for 2 days. Sections were cut at 70–80 nm thickness using an Ultracut S ultramicrotome and stained with Reynolds’ lead citrate and uranyl acetate. Sections were viewed and images recorded on a Tecnai T12 microscope.

### Quantification and statistics

#### ImageJ.

Mitochondrial morphology was manually assessed using ImageJ software (NIH). A scale was set by normalizing the pixel distance to the scale provided (nm) on each image (Supplemental Figure 16A). Area measurements were calculated by freehand selection around every whole mitochondrion observed in each image (Supplemental Figure 16B). Three separate measurements were taken; the mean value was used as the area. Diameter measurements were calculated by freehand lines at the largest part of every whole mitochondrion observed in each image (Supplemental Figure 16C). Mitochondrial numbers were manually counted in each image, which corresponded to a 4.95 μm^2^ (ex vivo) or 37 μm^2^ (in vitro) area of the RPE; only whole mitochondria were counted (Supplemental Figure 16D). Mitochondria were manually characterized into (Supplemental Figure 16D) “short tubular,” (Supplemental Figure 16E) “fragmented,” or (Supplemental Figure 16F) “long tubular” phenotypes.

#### Seahorse analysis.

As the number of wells used of a seahorse plate vary between experiment, the number of technical repeats (wells used) is reported in each experiment. The mean of these technical repeats (wells used) was subsequently used to provide the biological repeat for that experiment. If no technical repeats were used (only 1 well per condition), that sole value is used for the biological repeat of that experiment. The reported data are of biological repeats from at least 3 independent experiments.

#### Statistics.

Data are presented as mean ± SD for technical replicates or mean ± SEM for biological replicates. Statistical analysis was performed using an unpaired 2-tailed Student’s *t* test or 1-way ANOVA as specified for comparison between groups. *P* < 0.05 was considered significant.

### Study approval

All procedures were conducted under the regulation of the United Kingdom Home Office Animals (Scientific Procedures) Act 1986 and followed the ARVO statement for the use of animals in ophthalmic and vision research. The methods were carried out in accordance with the approved University of Bristol institutional guidelines, and all experimental protocols under Home Office Project Licence 30/3281 were approved by the University of Bristol Ethical Review Group.

## Author contributions

Conceptualization was done by ST and ADD; methodology was designed by ST and LMS; investigation was performed by LMS, EEV, NH, CN, NJ, EL, and ST; writing of the original draft was performed by ST, LMS, and ADD; review and editing of the draft were performed by MC, APH, EEV, NH, JL, CN, and EL; funding acquisition was done by ST and ADD; resources were provided by MC, EEV, NJ, EL, and CN; project management was done by ST; and supervision was provided by ST and ADD.

## Supplementary Material

Supplemental data

Supplemental Table 1

## Version 1 04/22/2021

Electronic publication
